# An IMS/ATP Assay for the Detection of *Mycobacterium tuberculosis* in Urine

**DOI:** 10.1155/2012/292605

**Published:** 2012-05-09

**Authors:** Dawn M. Hunter, Daniel V. Lim

**Affiliations:** Department of Cell Biology, Microbiology and Molecular Biology, University of South Florida, 4202 E. Fowler Avenue, ISA 2015, Tampa, FL 33620-7115, USA

## Abstract

*Background*. Although sputum smears are the gold standard for diagnosis of tuberculosis, sensitivity in HIV/TB coinfection cases is low, indicating a need for alternative methods. Urine is being increasingly evaluated. *Materials and Methods*. A novel method for detecting *Mycobacterium tuberculosis* (MTB) in synthetic urine using a combined IMS/ATP assay was evaluated. Preliminary work established standard ATP conditions and the sensitivity and specificity of the MTB antibody. Eighty-four blinded samples in four replicate assays were evaluated for the presence of MTB using labeled immunomagnetic beads for capture. Beads were separated, washed, and resuspended in broth and added to a microtiter plate. Bioluminescent output was measured and signal-to-noise ratios were calculated. All samples were plated on Middlebrook 7H10 agar or trypticase soy agar to determine limit of detection and recoveries. *Results and Conclusions*. MTB was distinguished from common bacteriuria isolates and other nontarget bacteria by its ATP results. IMS/ATP successfully detected 19 of 28 samples of MTB in synthetic urine with a limit of detection of 10^4^ CFU/ml. Sensitivity and specificity were 67.9% and 82.1%, respectively. This assay offers a possible rapid screening method for HIV-positive patients with suspected coinfection to improve MTB diagnosis.

## 1. Introduction

There are over 8 million new cases of tuberculosis (TB) annually, with increasing incidence in areas where HIV is prevalent [1]. In 2009, there were 9.4 million new TB cases, with 1.1 million among HIV-positive individuals [2]. Sputum smear microscopy remains the standard for diagnosis. However, sensitivity varies even among HIV-negative patients, with an average sensitivity of less than 60%, and is as low as 20% for patients with HIV/TB coinfection [3]. This is further complicated by an inability to produce sputum among HIV-positive individuals [4–6]. In addition to smear-negative pulmonary TB, HIV-positive individuals tend to have abnormal chest X-rays and clinical presentations, so diagnosis and treatment are often delayed [3, 6, 7]. Furthermore, the sputum procedure is limited in diagnosing extrapulmonary infection, which is more common among patients in this group [3, 8, 9]. The impact of smear-negative disease on diagnostics is significant; even nucleic acid amplification tests such as the Gen-Probe MTD and Roche Amplicor MTB, which have sensitivities greater than 95% in smear-positive cases, have reduced sensitivities of 40–77% in smear-negative cases [3, 10].

Extrapulmonary TB and disseminated disease are more likely with advanced immunosuppression [8, 10]. The kidneys may become involved due to the spread of bacilli through the vascular system from foci in the lung [1, 4]. MTB bacilli can be excreted through the kidneys and detected in the urine of patients who have no symptoms of genitourinary involvement [9, 11]. However, although it has been known since the 1960s that MTB can be found in the urine of patients with pulmonary TB, urine has been a less reliable clinical sample compared to sputum. Urine has not been recommended for routine diagnosis because the sensitivity of urine smear microscopy and the yield of urine cultures have been low; conventional diagnostic methods using urine samples have had “limited clinical usefulness” [4].

However, urine has been increasingly evaluated as a diagnostic sample due to recent developments enabling the detection of mycobacterial DNA and metabolic products in urine, particularly among HIV-infected patients [5, 9, 12–14]. While the number of bacilli in urine varies and excretion is intermittent, the bacillary load for HIV/TB patients with disseminated disease may be high [9, 15]. Mycobacteriuria may be more prevalent than historically believed based on urine culture alone, as recent studies have demonstrated PCR-positive urines with sensitivities up to 66.7% in smear-negative TB [6, 9]. Additionally, urine samples have advantages relative to other biological samples because bacilli can be easily concentrated [15–17], samples are easy to collect, and there are fewer risks associated with handling [4, 6, 9].

More pressing than the need for alternative diagnostic sample types is the need for rapid, easy-to-perform tests that can be used in a point-of-care format [3, 4]. There are several disadvantages to sputum smear microscopy: (1) it is not useful where there is a low bacillary load, (2) it is not useful in cases of extrapulmonary TB, and (3) microscopy can detect other acid-fast bacteria [18]. As already noted, smear-negative TB and extrapulmonary TB are more common in HIV-positive patients. Peripheral laboratories rely on microscopy methods, while no diagnostic tests are currently available at health posts [19]. As noted by the World Health Organization, diagnostic limitations have been a “crucial barrier” to meeting the challenges of HIV-associated TB [20]. 

Immunomagnetic separation (IMS) has been successfully used to concentrate and recover pathogenic mycobacteria, including MTB [21, 22]. IMS also enables specific target capture and decreases particulate interference in detection assays [23, 24]. ATP bioluminescence assays have demonstrated utility in bacteriuria screening [25, 26], quality control of BCG vaccines [27], and MTB antibiotic susceptibility testing [28]. Combining immunocapture with an ATP-based cell viability assay can provide rapid, specific, semiquantitative detection of live cells [29–31]. The method presented here combines IMS with an ATP-based cell viability assay to provide rapid, specific detection of MTB in urine. The method is easy to perform and could have use in settings where the rate of HIV/TB co-infection is high.

The objectives of this work were to study (1) the sensitivity and specificity of the MTB antibody, (2) the ATP amount released from MTB relative to other organisms using a standard ATP assay, (3) the effect of incubation time and urine pH on IMS/ATP results, and (4) the possible detection of MTB in synthetic urine.

## 2. Materials and Methods

### 2.1. Antibodies

Affinity-purified rabbit polyclonal antibody to MTB (BIODESIGN International, Saco, ME) in phosphate-buffered saline (PBS) and affinity-purified goat anti-rabbit antibody conjugated to horseradish peroxidase in PBS (Kirkegaard & Perry Laboratories, Inc., Gaithersburg, MD) were used as detection antibodies in ELISA. Biotin-labeled rabbit polyclonal antibody to MTB in PBS (BIODESIGN International, Saco, ME) was used as the capture antibody in the IMS/ATP assay. The antibody was immobilized on M280 Streptavidin Dynabeads (Invitrogen, Carlsbad, CA) according to the manufacturer's protocol. Antibody-labeled beads were stored for up to one week at 4°C until use.

### 2.2. Bacteria

MTB or *Mycobacterium tuberculosis* ATCC 25177 was the target microorganism for all assays. Nontarget microorganisms included common urine isolates [26], other mycobacteria, and *Candida *because of their morphological similarities to MTB. These strains were either ATCC strains or obtained from the University of South Florida Advanced Biosensors Laboratory (ABL) collection and included *Mycobacterium smegmatis* ABL 539, *Mycobacterium avium* ATCC 25921, *Mycobacterium intracellulare* ATCC 13950, *Mycobacterium gordonae* ATCC 14470, *Rhodococcus rhodochrous* ABL 538, *Candida albicans* ABL 537, *Staphylococcus aureus* ATCC 25923, *Staphylococcus epidermidis* ATCC 12228, *Staphylococcus simulans* ATCC 11631, *Enterococcus faecalis* ATCC 19433, *Escherichia coli* K12 ABL 552, *Pseudomonas aeruginosa* ATCC 15442, and *Klebsiella pneumoniae *ATCC 29019. Working stocks of nonmycobacterial strains prepared from frozen cultures were grown for 18 h at 37°C in tryptic soy broth (TSB; BD, Franklin Lakes, NJ). Working stocks of mycobacteria prepared from frozen cultures were grown for two days to three weeks (depending on the strain) at 37°C and 5% CO_2_ on Middlebrook 7H10 agar (BD, Franklin Lakes, NJ). All working stocks were maintained at 4°C for up to 30 days. A fresh culture of MTB was incubated on a weekly basis for use in assays. Other bacterial strains used were grown overnight on tryptic soy agar (BD, Franklin Lakes, NJ) at 37°C.

### 2.3. Sample

Synthetic urine was purchased from Ricca Chemical Company (Arlington, TX). Synthetic urine has been used in a variety of studies, including method validation studies [32–34]. The urine contains urea, sodium chloride, magnesium sulfate heptahydrate, calcium chloride dihydrate, and water. Urine pH was adjusted from *∼*8.4 to 7.1 ± 0.1 or 5.5 ± 0.1 to determine whether urine pH influenced IMS. Based on these results, urine pH was adjusted to 7.1 ± 0.1 for all blinded assays (described in what follows).

### 2.4. ELISA

Cells were suspended in 0.01 M PBS with 0.1% Tween-80 (PBST80). Glass beads (0.1 mm, BioSpec Products, Inc., Bartlesville, OK) were added to mycobacterial suspensions to aid in dispersion. Cell suspensions were serially diluted (1 : 10) in PBST80, and consecutive serial dilutions were added to MaxiSorp 96-well microtiter plates (Nalge Nunc International, Rochester, NY) in triplicate (100 *μ*L/well) and incubated for 1 h at 37°C. Plates were washed 3 times with PBS containing 0.05% Tween-20 (PBST), then coated with 100 *μ*L primary antibody in blocking buffer and incubated for 30 minutes at 25°C. Plates were washed again, 100 *μ*L of HRP-labeled secondary antibody in blocking buffer was added to each well, and plates were incubated for 30 min at 25°C. Plates were washed a final time, and peroxidase activity was detected using a QuantaBlu kit (Thermo Fisher, Item 15169) according to manufacturer instructions. Plates were read on a SpectraMax Gemini XS with the following parameters: 340 nm excitation, 470 nm emission, 455 nm cutoff, and PMT set to auto.

Each organism was assayed in duplicate. Signal-to-noise ratios (S : N) for each strain at each concentration were determined by dividing the raw fluorescence by the average background fluorescence. Triplicate S : N from duplicate plates were averaged and standard deviations were calculated. Average S : N greater than or equal to 2.0 were considered positive.

### 2.5. Standard ATP Assays

Cells were suspended in Mueller Hinton II broth (MHII) containing 0.1% Tween-80 (MHII-80) and serially diluted (1 : 10) in MHII-80. Consecutive serial dilutions were added in triplicate (100 *μ*L/well) to Lumitrac 600 microtiter plates (BioExpress, Kaysville, UT). MHII-80 without cells was used to establish background. BacTiter-Glo reagent was added to each well (100 *μ*L/well), contents were mixed briefly on an orbital shaker, and bioluminescent output was measured on a GloMax 96 luminometer (Promega, Madison, WI) at 5, 10, 15, 20, and, in some assays, 40 minutes. S : N for each strain at each concentration were determined by dividing the raw fluorescence by the average background fluorescence.

### 2.6. IMS/ATP Assays

Initially, standard IMS/ATP assays were completed using MTB suspended in PBST80. Twenty microliters of antibody-labeled beads were added to each sample, which were then incubated with shaking for 60 minutes at 37°C. Beads were separated from the sample using a magnet, the sample was removed, and beads were washed three times with PBST then resuspended in MHII broth. MHII broth containing only labeled beads was used to establish background. Samples (100 *μ*L per well) were added to Lumitrac 600 plates followed by 100 *μ*L per well of BacTiter-Glo reagent. Contents of the plates were mixed briefly on an orbital shaker, incubated for 5 min at 25°C, and read at 5, 10, 15, and 20 minutes using a GloMax 96 luminometer with no delay and 1 sec integration. Sample S : N were determined by dividing the raw fluorescence by the average background fluorescence.

Subsequently, the IMS/ATP assay was evaluated using MTB suspended in synthetic urine. The procedure was the same except sample incubation time was 30–60 min at 37°C to determine the impact of incubation time on IMS/ATP results. For the final set of IMS/ATP assays in synthetic urine, sample incubation time was 30 min, and samples were blinded and number coded. For all blinded samples, four replicate wells at each concentration were averaged for each time point. Sample codes were revealed after assay completion and analysis.

### 2.7. Statistical Analysis

To evaluate the effect of urine pH and incubation time, paired *t*-tests were performed comparing S : N at each concentration from 10^3^ to 10^6^ CFU/mL at each pH or for each incubation time (SigmaPlot 11, Systast Software, Inc., Chicago, IL). Differences in means were considered statistically significant for *P *≤ 0.05 (95% confidence level).

## 3. Results and Discussion

### 3.1. ELISA

The detection limit of MTB by ELISA was approximately 10^5^ CFU/mL. Some cross-reactivity with *M. smegmatis*, *E. coli* K12, *K. pneumoniae*, and *P. aeruginosa* was noted, but S : N for these strains ranged from 2.5 to 3.4 RLU, slightly above the positive cutoff of 2 RLU. There was increased cross-reactivity for these strains at concentrations near 10^7^ CFU/mL; therefore, the assay would be utilized for screening purposes rather than confirmation due to the potential for false positives. Also, the relatively high detection limit of the assay would not positively identify low bacillary load samples. However, with the standard practice of centrifugation, filtration, and/or pooling of urine samples combined with IMS, the numbers of tubercle bacilli could be in the detectable range.

### 3.2. Standard ATP Assays

**Figure 1:**
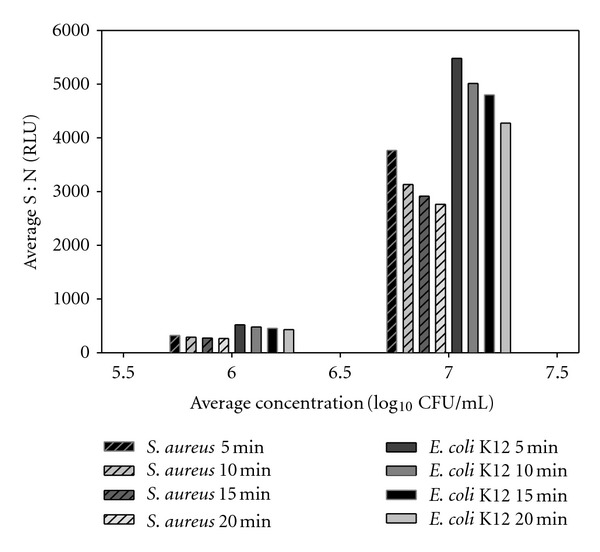
Standard ATP assay results for *S. aureus* and *E. coli* K12 at 10^6^ and 10^7^ CFU/mL. Organisms were suspended and serially diluted in Mueller Hinton II broth containing 0.1% Tween-80 and evaluated using the BacTiter-Glo basic ATP assay.

**Figure 2:**
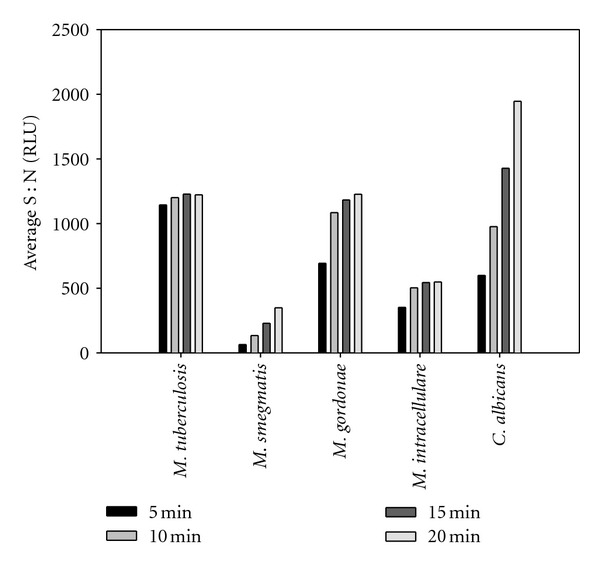
Standard ATP assay results for various mycobacteria and *Candida albicans* at approximately 10^6^ CFU/ml. Organisms were suspended and serially diluted in Mueller Hinton II broth containing 0.1% Tween-80 and evaluated using the BacTiter-Glo basic ATP assay.

Generally, Gram-positive and Gram-negative bacteria exhibited decreasing signal over time as ATP was consumed (Figure 1). In contrast, organisms with a thicker cell wall, including the mycobacteria and *Candida*, exhibited increasing signal over time due to the additional time required to break down the cell walls to release ATP (Figure 2). The increase in signal is a distinguishing feature of mycobacteria and *Candida* that could allow differentiation from other common organisms associated with bacteriuria, namely, *E. coli* and mixed Gram-positive cocci [26]. It was also routinely observed that MTB had a modest increase in signal relative to other mycobacteria. The signal at 20 min was 0.96–1.06 times higher than the signal at 5 min for MTB, whereas the signal at 20 min for *M. smegmatis*, *M. gordonae*, and *M. intracellulare* was 1.65–3.26, 1.19–1.80, and 1.20–1.55 times higher, respectively, than the signal at 5 min. The signal for *C. albicans* at 20 min was 2.8–3.2 times higher than the signal at 5 min. The modest increase in MTB signal could enable differentiation between MTB and other mycobacteria or *C. albicans* in a screening assay. It should be noted, however, that these results may not mirror real-world samples. *C. albicans* was grown for 18 h and used immediately, whereas the mycobacteria were grown for 2 days (*M. smegmatis*) up to 3 weeks (MTB), so the metabolic activity of the mycobacteria varied in comparison and could have been lower due to differences in growth phase.

Furthermore, only evaluating samples for a change in S : N over time would lead to misidentification of samples because of noted cross-reactivity in ELISA and similar increases in S : N for other mycobacteria and *C. albicans*. The standard ATP results for MTB revealed that, generally, at least one time point after the first time point (T1) will be greater than T1 and that the average S : N over all four time points should be greater than T1 due to the increases in S : N. Therefore, data for the blinded assays were evaluated on two factors: (1) whether the S : N at any time point after T1 was greater than T1 and (2) whether the average S : N over all four time points divided by T1 was greater than 1. For this analysis, because MTB demonstrated increasing S : N over time, factor (2) was only evaluated if there was an increase in S : N after T1; otherwise, the sample was considered negative. 

### 3.3. IMS/ATP Assays

#### 3.3.1. Initial Assays

**Figure 3:**
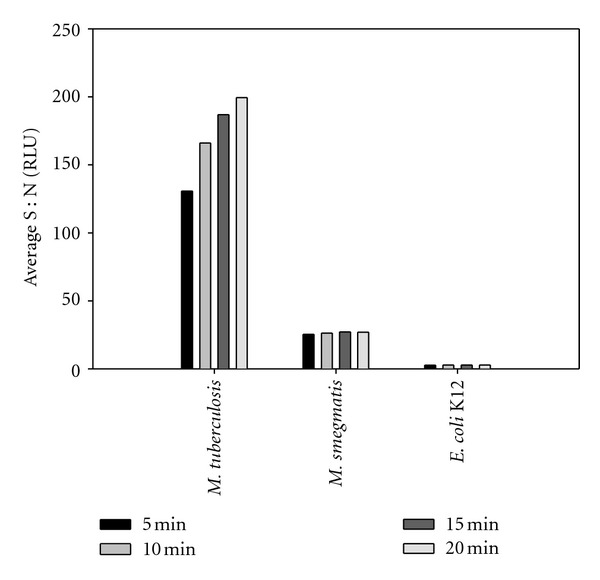
IMS/ATP assay at approximately 10^6^ CFU/ml in PBS containing 0.1% Tween-80.

IMS/ATP parameters were established through preliminary assays. Standard IMS assays in PBST80 revealed a clear affinity of the MTB antibody for MTB over nontargets using IMS (Figure 3), which is significant given the cross-reactivity observed in ELISA. Additionally, side-by-side IMS/ATP assays performed using urine at pH 5.5 ± 0.1 and 7.1 ± 0.1 revealed that there was no significant difference among S : N and that S : N were in the same log for the same concentration regardless of pH. Recoveries as determined by total viable counts were also similar (data not shown). Based on these data coupled with normal human urine normally being close to pH 7 [35], all subsequent assays were performed at pH 7.1 ± 0.1.

There was no significant difference in mean S : N for either 30 or 60 min incubation time, with mean S : N for MTB being slightly higher with 30 min incubation, while mean S : N for *E. coli* K12 were slightly higher with a 60 min incubation. Increased incubation time may increase the potential for nontarget binding while failing to improve detection of the target. Therefore, a 30 min incubation period was implemented for all subsequent assays. There were several advantages to this approach: (1) antibody-antigen binding is rapid and strong, and a 60 min incubation period may be unnecessary, (2) a short incubation period would not increase the metabolic activity or enhance ATP levels for slow-growing MTB as would be expected with an organism that has a shorter generation time, and (3) overall assay time was reduced.

#### 3.3.2. Blinded Assays

One blinded assay including 20 samples (4 MTB and 16 nontargets) was performed to evaluate the sample analysis described in Section 3.2. All four MTB samples were identified using this analysis. In addition, a cutoff for factor (2) was identified based on the results for the nontargets. The mean for average S : N/T1 was 0.95 ± 0.2, making the upper limit for negative samples 0.97. Therefore, if samples met factor (1), they were considered positive for average S : N/T1 ≥ 0.98. Four replicate assays were completed using this analysis. The results for MTB are reported in Table 1.

Overall, 19 of 28 MTB samples were flagged for confirmation, resulting in a sensitivity of 67.9%. This is much higher than the approximately 20% sensitivity typical of sputum smear microscopy in HIV-positive patients with suspected TB [26] and is on a par with alternative diagnostic tests like the LAM ELISA, which has a reported sensitivity of 67–85% in HIV-positive patients [5]. All 19 flagged samples exhibited an increase in S : N for at least one time point after T1, and all yielded an average S : N/T1 of 0.98 or greater. None of the samples considered negative exhibited an increase in S : N for any time point after T1, and this result was not restricted by concentration. One of one sample at 10^3^ CFU/mL, three of seven at 10^4^, four of ten at 10^5^, and one of ten at 10^6^ were negative by this analysis. The variability in results could have been impacted by several factors, including the age of the cultures, whose metabolic activity may have been significantly lower than what would be observed for bacteria isolated from an active infection, as well as the use of spiked, synthetic urine. It would be important to evaluate this method using actual clinical samples to determine whether these factors have, in fact, influenced the results.

 The specificity of the assay was 82.1%, with 10 false-positives out of 56 total samples (Table 2). All of the false-positives met both factors of the analysis—they exhibited an increase in S : N at any time point after T1, and they yielded an (average S : N/T1) > 1.0 in all instances. Two organisms—*K. pneumoniae* and *E. faecalis*—satisfied factor (1) in three instances, but none satisfied factor (2), and thus were considered negative. As for *M. smegmatis*, the false positive was not surprising given the cross-reactivity noted at 10^6^ CFU/mL in ELISA. However, *M. smegmatis* is rarely found in urine and not at the concentrations evaluated [36, 37]. Therefore, *M. smegmatis* was not evaluated in replicates 3 and 4.

Two observations can be made for *C. albicans*. First, standard ATP assays revealed that *C. albicans* generally yields higher overall signals and greater increases relative to MTB. This is evident from the S : N at T1 (Table 2), as well as the results for average S : N/T1 relative to MTB. The mean average S : N/T1 for *C. albicans* was 1.47 versus 1.14 for positive MTB samples. Second, *C. albicans* is not as commonly isolated from urine specimens as other organisms, so it is less likely to pose a problem with real-world samples. Yeasts comprised only 4 of 178 samples (0.02%) in one study of microorganisms isolated from 400 urine specimens [26].

Finally, the only other organism to result in a false-positive was *M. intracellulare*. This result is significant since *M. intracellulare* is part of the *M. avium* complex known to be an opportunistic pathogen of immunocompromised patients [38]. While only one of seven *M. intracellulare* samples and none of five *M. avium* samples resulted in a false-positive, it would be important to evaluate these organisms further in future assays.

The mean S : N across replicates for a given concentration were consistent, with S : N at 10^4^ and 10^5^ CFU/mL in the same log (Table 3). An increase in S : N over time was observed for the two highest concentrations but not for the samples at 10^4^ CFU/mL, which may explain the inconsistent detection of this concentration in individual assays.

The described IMS/ATP assay could have utility as a screening assay in reference or peripheral laboratories where HIV/TB co-infection rates are high. The assay is rapid, takes less than 1 h to complete, and requires minimal reagents and equipment. Even with potential cross-reactivity, the assay has merit, because the objective is to screen, rather than confirm. This novel assay is potentially useful as a diagnostic, screening assay for the detection of MTB in HIV-positive patients.

## Figures and Tables

**Table 1:** Summary results for detection of MTB in synthetic urine—blinded assays.

Replicate	Conc. (CFU/mL)	S : N at T1	S : N at any time point > T1	Average S : N (T1–T4)	Average S : N/T1	Send for confirmation
1	10^5^	17.60	X	20.40	1.16	Yes
	10^5^	12.69	X	18.45	1.45	Yes
	10^5^	15.81	X	18.11	1.15	Yes
	10^4^	2.14		2.07	0.97	No
	10^4^	1.64	X	1.72	1.05	Yes
	10^4^	2.30	X	4.09	1.78	Yes
	10^3^	1.02		0.98	0.96	No

2	10^6^	9.86	X	9.75	0.99	Yes
	10^6^	12.99	X	12.83	0.99	Yes
	10^6^	11.27	X	11.44	1.01	Yes
	10^5^	4.00		3.76	0.94	No
	10^5^	2.71		2.65	0.98	No
	10^5^	3.61	X	3.58	0.99	Yes
	10^4^	12.47	X	12.24	0.98	Yes
	10^4^	15.43		15.05	0.98	No

3	10^6^	4.45	X	4.60	1.03	Yes
	10^6^	3.83		3.74	0.98	No
	10^6^	3.72	X	3.75	1.01	Yes
	10^5^	1.64	X	1.64	1.01	Yes
	10^5^	1.16		1.11	0.95	No
	10^5^	1.23		1.19	0.97	No
	10^4^	1.11	X	1.09	0.98	Yes
	10^4^	1.12		1.09	0.97	No

4	10^6^	1.71	X	1.93	1.13	Yes
	10^6^	8.57	X	14.65	1.71	Yes
	10^6^	1.39	X	1.40	1.01	Yes
	10^6^	5.62	X	7.20	1.28	Yes
	10^5^	1.65	X	1.67	1.01	Yes

**Table 2:** Summary results for false positives in the blinded assays.

Organism	Replicate	Conc. (CFU/mL)	S : N at T1	S : N at any time point > T1	Average S : N (T1–T4)	Average S : N/T1	Send for confirmation
*M. smegmatis*	1	10^6^	23.80	X	57.50	2.42	Yes
	2	10^6^	4.29	X	4.52	1.05	Yes
	2	10^6^	3.98	X	4.26	1.07	Yes

*M. intracellulare*	3	10^6^	1.56	X	1.56	1.00	Yes

*C. albicans*	3	10^6^	347.1	X	378.0	1.09	Yes
	3	10^6^	264.2	X	392.4	1.49	Yes
	3	10^5^	44.76	X	48.48	1.08	Yes
	4	10^5^	15.81	X	28.14	1.78	Yes
	4	10^5^	19.03	X	32.6	1.71	Yes
	4	10^4^	1.75	X	2.92	1.67	Yes

*K. pneumoniae*	3	10^6^	1.09	X	1.05	0.96	No

*E. faecalis*	3	10^6^	7.95	X	7.73	0.97	No
	4	10^6^	13.54	X	12.87	0.95	No

**Table 3:** Mean S : N for detection of *M. tuberculosis* by IMS/ATP. Data is averaged from the four replicates reported.

Mean concentration (CFU/mL)		2.82*E* + 06	2.82*E* + 05	2.82*E* + 04
Mean S : N	5 min	8.74	2.16	1.16
	10 min	10.01	2.25	1.15
	15 min	11.26	2.39	1.13
	20 min	12.46	2.43	1.11
